# Enhancing care: evaluating the impact of True North Sexual Health and Rehabilitation eTraining for healthcare providers working with prostate cancer patients and partners

**DOI:** 10.1093/sexmed/qfae033

**Published:** 2024-06-14

**Authors:** Andrew G Matthew, Deborah McLeod, John W Robinson, Lauren Walker, Richard J Wassersug, Stacy Elliott, Steven Guirguis, Taylor Incze, Lianne Trachtenberg

**Affiliations:** Department of Surgical Oncology, Princess Margaret Cancer Centre, University Health Network, Toronto, ON M5G 1Z5, Canada; School of Nursing, Dalhousie University, Halifax, NS B3H 2Y9, Canada; Department of Oncology, University of Calgary, Calgary, AB T2N 1N4, Canada; Department of Oncology, University of Calgary, Calgary, AB T2N 1N4, Canada; Cellular and Physiological Sciences, University of British Columbia, Vancouver, BC V5Z 1M9, Canada; BC Center for Sexual Medicine, Vancouver Coastal Health Authority, Vancouver, BC V6Z 2K5, Canada; Department of Surgical Oncology, Princess Margaret Cancer Centre, University Health Network, Toronto, ON M5G 1Z5, Canada; Department of Surgical Oncology, Princess Margaret Cancer Centre, University Health Network, Toronto, ON M5G 1Z5, Canada; Department of Surgical Oncology, Princess Margaret Cancer Centre, University Health Network, Toronto, ON M5G 1Z5, Canada

**Keywords:** sexual dysfunction, oncosexology, sexual health, prostate cancer, online education, survivorship

## Abstract

**Background:**

Educational programs that enhance healthcare providers’ competence in managing the care of patients with sexual dysfunction following prostate cancer treatments are needed to facilitate comprehensive sexual health treatments for patients and their partners.

**Aim:**

In this study we evaluated the impact of a real-world online sexual health educational intervention called the True North Sexual Health and Rehabilitation eTraining Program. This program is designed to increase healthcare providers’ knowledge and self-efficacy in providing sexual healthcare to prostate cancer patients and their partners.

**Methods:**

Healthcare providers were invited to join a 12-week virtual training program. Participants completed precourse surveys (n = 89), retrospective prepost surveys (n = 58), and a 3-month follow-up survey (subset n = 18) to assess retention of relevant outcomes. Additionally, a course satisfaction survey was administered to participants (n = 57) at the end of the course.

**Outcomes:**

The main outcomes focused on participants’ perceived knowledge and self-efficacy in conducting assessments and providing interventions for various relevant physical, functional, psychological, and relational domains of sexual dysfunction in prostate cancer patients and their partners.

**Results:**

According to the retrospective analysis of post-then-pre–survey results, graduates perceived that their knowledge of and self-efficacy in providing sexual health counseling improved after completing the course. The 3-month follow-up survey indicated that the course graduate self-efficacy remained high 3 months after the course. Furthermore, the satisfaction survey indicated that a vast majority (98.2%) of participants were satisfied with the educational intervention.

**Clinical Implications:**

This real-world sexual health educational intervention can increase self-efficacy and knowledge in healthcare providers who are supporting prostate cancer patients dealing with sexual dysfunction.

**Strengths and Limitations:**

The use of a retrospective post-then-pre–survey helped to mitigate response shift bias while minimizing data gaps. However, it is important to note that this investigation was not a traditional research study and lacked a control group, thus limiting causal attributions.

**Conclusion:**

The True North Sexual Health and Rehabilitation eTraining program acts as an accessible and effective resource for healthcare providers seeking specialized training in providing sexual healthcare for prostate cancer patients and their partners.

## Introduction

Prostate cancer (PCa) and its treatments introduce a wide range of sexual health complications that remain insufficiently addressed in the healthcare system.^1–4^ Patients and their partners report a need for support, counseling, and education for cancer-related sexual dysfunction.^1^^,^^3^^,^^5^ In fact, patients cite sexual health concerns as their most significant unmet need following PCa treatment.^6–8^ Patients with PCa are often dissatisfied with the lack of information provided by their healthcare providers (HCPs), such as oncologists, urologists, and nurses, regarding anticipated treatment responses and treatment side effects.^9^ Similarly, patients report inadequate treatment and counseling for sexual dysfunction in the oncology setting. Although men diagnosed with PCa report interest in receiving support and information, as many as 40% do not seek professional support for their PCa-related concerns due to individual and systemic barriers.^8^^,^^9^ The preferred source of information for patients and their partners is counseling from their HCP.^8–10^ Although many men reportedly prefer to receive help from urologists, they are open to help from other HCPs.^10^

Unfortunately, many HCPs, including oncologists, nurses, psychologists, and social workers lack the necessary skills and expertise to address sexual dysfunction in patients who have undergone cancer treatment.^11^^,^^12^ Despite the training they have received on patients’ sexual health issues, HCPs report a lack of confidence and inadequate knowledge to recommend a course of treatment confidently. Some HCPs report feeling uncomfortable discussing sexual health with older patients, believing it is inappropriate^12^. However, many HCPs have expressed interest in acquiring additional training in sexual health counseling to bridge this knowledge gap.^11^

Overall, this situation highlights the need for educational programs that enhance HCP competence in sexual health counseling of patients who have undergone PCa treatment and their partners. Such educational programs can improve patient–HCP interactions and facilitate comprehensive sexual dysfunction treatment, recommendations, and programming. A survey by Schnur and Montgomery (2012) found that HCPs prefer to participate in online training vs in-person education programs,^13^ and such programs can be effective in training HCPs.^14^^,^^15^

To address this gap in care, a multidisciplinary Canadian team of psychologists, nurses, researchers, and uro-oncologists developed an “expert-consensus” course, True North Sexual Health and Rehabilitation eTraining (SHAReTraining),. This online course was crafted to educate HCPs in providing PCa-specific sexual health counseling that employs a biopsychosocial approach devised by a multidisciplinary team. In this report, we detail the influence of the SHAReTraining program on bolstering perceived knowledge and self-efficacy of HCPs in evaluating and managing sexual dysfunction in men/couples after PCa treatment. The SHAReTraining program goes beyond knowledge dissemination, incorporating methods to augment self-efficacy as a crucial element in nurturing HCP proficiency. Self-efficacy is the belief of an individual in their capability to perform a specific task and is a powerful predictor of their performance.^16^ Together, knowledge and self-efficacy serve as the foundation of competence, equipping individuals with the skills and confidence to engage in sexual healthcare. Although this investigation of SHAReTraining deviates from conventional research studies, the impact of this program reported here was assessed through a practical real-world lens.

## Objectives

In this evaluation we aimed to examine the impact of a real-world online sexual health educational intervention in improving HCP knowledge and self-efficacy in providing sexual healthcare to PCa patients and their partners.

## Methods

### Design

The 12-week virtual SHAReTraining program utilized a real-world approach for training HCPs to provide sexual healthcare to PCa patients nd their parthers. Research ethics approval (No. 22-5679) was granted for a retrospective evaluation of the SHAReTraining program. At baseline, all participating HCPs completed a demographics survey, providing information on current job title, professional experience in oncology, and motivation for enrolling in the course. Immediately following the course, participants completed a retrospective post-then-pre (RPP) course evaluation to assess changes in their perceived knowledge and self-efficacy in sexual health counseling. The evaluation of the impact of the program was guided by the typology of learning described in Kirkpatrick and Kirkpatrick (1994),^17^ with a focus on the acquisition of learning, and the logic model used to evaluate the Canadian Interprofessional Education for Collaborative Patient Centered Practice.^18^ Additionally, participants completed a self-report measure assessing their satisfaction with the course instructor, content, and technological accessibility. Participants in the first phase of the pilot (cohorts 1-3) were invited to complete a 3-month follow-up evaluation which examined participants self-efficacy.

### Course setting

The virtual SHAReTraining course was initially hosted on the Interprofessional Psychosocial Oncology Distance Education (IPODE) online training platform (cohorts 1-8). Later, prompted by the retirement of a key team member from the IPODE and SHAReTraining team, the platform was transitioned to the de Souza Institute, which was identified as a more suitable long-term host. The relocation of the course to the de Souza Institute has provided SHAReTraining with a permanent and adaptable infrastructure, facilitating seamless modifications and maintenance as required. Importantly, there are no fundamental differences between the two online platforms in terms of user-friendliness, cost, accessibility, and other essential factors.

### Recruitment

Recruitment efforts were designed to target a diverse audience, including graduate students and practicing HCPs. The course was advertised through various channels, including IPODE and the course listings of de Souza Institutes and professional affiliations such as the Canadian Association of Psychosocial Oncology (CAPO). Moreover, team members identified contacts within each province to recruit and market the course, promoting its availability to healthcare communities and PCa cancer centers across Canada. Last, SHAReTraining used snowball sampling, which contributed to program visibility beyond Canada and attracted international participants to the course.

### Participants

Participants who registered for the course were largely self-selected. These individuals were not actively recruited but rather motivated by their desire to enhance their knowledge of sexual health in the context of PCa. This self-selection approach was considered the most suitable method, given the comprehensive nature of the course, which demanded substantial time and commitment from each participant.

Eligibility for the course was extended to any regulated HCP or students holding a degree in a healthcare-related field. Notably, as ethical review was granted for retrospective evaluation and data was anonymized, informed consent was not deemed necessary, and participants were not provided compensation for their involvement.

This study was approved by the UHN Research Ethics Board, study ID: 22-5679. The procedures used in this study adhered to the tenets of the Declaration of Helsinki.

### Educational intervention

The SHAReTraining course was modeled after and developed using the following resources: (1) the existing online *Sexual Health and Cancer* courses offered through the IPODE and the British Columbia Institute of Technology; and (2) existing PCa-specific sexual health and rehabilitation clinical programs, namely, the Princess Margaret Cancer Centre Prostate Cancer Rehabilitation Clinic,^19^ and the Tom Baker Cancer Centre Prostate Cancer Sexuality and Bladder Rehabilitation Program.

Structured as a continuing education course, SHAReTraining included exploration of theory, research, and clinical practice in PCa and sexual health. The overarching objective was to nurture expertise in person/couple-centered care. A pedagogical focus of the training was centered on first-person illness narratives. Interviews and presentations were used to help participants understand the sexual health and cancer experience from the perspective of patients and their partners.

Since the SHAReTraining program was not constructed as a traditional study intervention but rather as a real-world educational course, its practical educational value was always prioritized. As such, the content was updated regularly to reflect advancements in the field of PCa sexual health as well as feedback gathered from the participant satisfaction surveys.

The SHAReTraining program spanned 12 weeks and featured a curriculum comprising weekly 1.5-hour live virtual seminars, readings, and discussion forums. To facilitate focused learning, the course curriculum was organized into six overarching topics (see Table 1). Participants were expected to attend all 12 live seminars and had to attend a minimal of 8 seminars to successfully complete the program. All sessions were recorded and accessible to participants on the learning platform.

**Table 1:** SHAReTraining Course Curriculum.

**Week 1-3: Introduction to sexual healthcare in cancer**
• Orientation to the course • The experience of prostate cancer • Rationale for sexual healthcare in prostate cancer • Enhancing skill, knowledge, and comfort
**Week 4: Models/theory of sexual health in couples**
• Philosophy of care in addressing sexual health • An integrated model of sexual health
**Week 5: assessment**
• Sexual health assessment • The PRISM model • Revisiting the PLISSIT model
**Week 6-10: Intervention**
• Resuming sexual activity (intercourse and outercourse) • Maintaining intimacy (closeness and connection) • Addressing the needs of gay and bisexual men • Erectile rehabilitation • Proerectile therapies • ADT and sexual function
**Week 11: Professional ethics and issues**
• Self-care and ethics in the context of providing sexual healthcare • Case study presentations
**Week 12: Implementing sexual health programming in a healthcare setting**
• Leadership and change theory in the context of providing sexual healthcare • Project presentations • What it means to be a site champion

In terms of facilitation, each cohort benefited from two facilitators—a lead and a cofacilitator. The facilitators delivered the live sessions while encouraging participant discussion. Initially, during the early cohorts the course facilitators were members of the study team who had actively contributed to the course design. However, as the course continued, additional facilitators were onboarded as needed. The minimal requirement for becoming a SHAReTraining facilitator included successful completion of the course, a minimum of 1 year of experience as a sexual health counselor, and cofacilitating the course alongside an experienced facilitator for 2 cohorts.

## Evaluation and measures

### Assessment of knowledge and self-efficacy in sexual health counseling

To gauge participants’ perceived knowledge and self-efficacy the research team developed an RPP survey, referred to as the Knowledge and Self-Efficacy Survey (KSE survey). Constructed based on the key topics within the SHAReTraining curriculum, the survey questions were directly linked to critical learning domains of the course. The KSE survey employed an RPP design that was similar to a pretest-posttest design but differed in that it was solely administered after course completion. This RPP has been used successfully to evaluate educational, social, and health science program outcomes and to avoided the need for a pretest assessment.^20^ Compared to traditional pretest-posttest assessments, The RPP design helps reduce the response shift bias typically observed in pretest evaluations.^20–23^ This bias is a result of the participants’ internal frame of reference changing over the course of an intervention, specifically, how the internal perceptions of participants can change as they learn and experience new things. In this intervention participants might initially believe that they have a high level of knowledge, but as they progress through the course, they gain new insights. By the end, they may realize that their understanding was not as comprehensive as they initially perceived. The RRP design allows participants to provide a more accurate reflection of their initial and current state because they have a clearer view of their starting point after going through the intervention. Completing both surveys successively helps control for any response shift bias by allowing participants to maintain a consistent internal frame of reference.

Furthermore, the RPA design minimizes incomplete data because it is administered successively postintervention.^23^^,^^24^ By conducting the survey after coursecompletion, participants are better equipped to gauge their knowledge levels and self-efficacy, allowing for a more informed self-assessment.^23^^,^^24^

Although the authors did not directly measure participants’ knowledge by relying on self-reported assessments, it is widely recognized that knowledge increases after course completion. Instead, the emphasis of this program was placed on evaluating participants’ self-efficacy, as it is less evident whether the SHAReTraining course could effectively enhance self-efficacy. Higher levels of self-efficacy are crucial because they can help HCPs feel more comfortable and willing to initiate discussions about sexual health with their patients. Additionally, RPP survey outcomes tend to have higher correlations with objective and performance measures than with traditional pretest-posttest assessments.^22^

The KSE survey encompassed 8 domains, totaling 138 items, rated on a 10-point Likert scale (1 = I have little knowledge/self-efficacy, 10 = I am very knowledgeable/confident). These items were developed by the research team and directly aligned with key learning areas of the course. The initial 78 items focused on assessing perceived knowledge, and the subsequent 60 items focused on gauging self-efficacy. The outcomes were divided into 2 main domains: (1) knowledge of sexual health counseling and PCa and (2) self-efficacy to initiate conversation about sexual health and provide information resources. Each domain included the following subdomains: Intervening with Sexual Issues, Intervening with Sexual Issues for Female/Male Partners, and Collaborating and Adjusting Counseling.

Sample items evaluating perceived knowledge included: “*I am knowledgeable about how to intervene with men experiencing erectile dysfunction” and “I am knowledgeable about how to intervene with men with low desire.”* Example items assessing self-efficacy included: *“I am confident about how to initiate conversations with patients about sexual health concerns”* and *“I am confident about how to provide appropriate instructions on the use of pro-erectile aids and devices.”*

Since the SHAReTraining program was not a traditional research study and participation was entirely voluntary, survey completion was also optional. Importantly, opting not to participate in the surveys did not affect participants’ ability to complete and graduate from the course. Furthermore, although the KSE survey had 138 items, the survey had a 93% completion rate and typically took 13 minutes to complete.

### Postcourse satisfaction survey

Following course completion, participants responded to a 16-item postcourse satisfaction survey. The research team developed this survey for this study. The survey featured both closed- and open-ended questions. Close-ended questions utilized a 5-point Likert scale (1 = Strongly Agree to 5 = Strongly Disagree) and assessed various aspects, including course content, technology, instructor performance, and overall satisfaction with the course. Meanwhile, open-ended questions explored participants’ learning experiences, suggestions for course improvements, desired course changes, and satisfaction with the web-based delivery format.

### Three-month follow-up survey

All graduates from the first 3 cohorts who completed the course (n = 23) were invited to participate in a follow-up survey, which was sent 3 months after course completion. Ultimately, (18 of the 23 participants completed the survey. The 3-month follow-up survey focused exclusively on the self-efficacy domain of the KSE survey, assessing current levels of self-efficacy. Notably, this survey did not assess perceived knowledge, as the primary interest of the research team was understanding how the SHAReTraining course influenced HCP actions and willingness to engage in discussions about sexual health, rather than evaluating knowledge retention. This survey aimed to provide insights into the levels of HCP confidence, which can serve as an indicator of their ability to translate knowledge into practical care.

### Data analysis

Descriptive analyses were conducted on study variables (eg, frequencies, measures of central tendency). Knowledge and self-efficacy scores were calculated by summing the total domain scores of each participant for each of the 8 domains. The mean (SD) domain scores were calculated for KSE survey outcome measures.

Paired-sample *t*-tests were used to assess the effects of the SHAReTraining course on pre- and postcourse perceived knowledge and self-efficacy levels. To account for multiple comparisons, we applied the Bonferroni adjustment, setting the significance level at α = .00625 per test (0.05/8). The acceptability and satisfaction criteria were met if 75% of participants rated the training program favorably (“agree”/“strongly agree” or “satisfied”/“very satisfied”) in the postintervention program evaluations. Data were analyzed using IBM SPSS Statistics (Version 24).

## Results

### Participant characteristics

Eleven cohorts participated in SHAReTraining between September 2015 and December 2022 for a total of 107 participants in the course. Most participants identified as female (87.6%), worked in an urban setting (91%), and reported less than 5 years of experience working with PCa patients (67.4%) or in the field of sexual health (76.4%). Approximately half of the participants were from Ontario, Canada (48.6%) and were predominantly nurses (45.8%). When asked about their motivation to take the course, 23.6% reported wanting to fill a gap in clinical care, 30.1% wanted formal training in this area, and 23.6% reported that the course was suggested by their employer. When asked what interested them most, 60.7% of participants reported course content, 5.6% reported delivery, and 19.1% cited both content and delivery. Most participants (89.9%) reported that their managers fully supported their involvement in the course; however, 2.2% reported that they did not have that support. Twelve participants (11.2%) withdrew from the course prematurely, citing the following reasons: (1) a sudden change in their schedule, (2) inability to balance personal life, work, the and course, and/or (3) lack of awareness of the time commitment required (see Table 2). The KSE survey was offered to all participants, except those who withdrew, with 61.0% of participants (n = 58) completing the survey.

**Table 2:** Summary of participants’ demographic information.

**Category**	**Subcategory**	**Values**
Age, y (n = 107)	Mean (SD)	41.27 (12.0)
	Range	24-68
Province, No. (%) (n = 107)	Ontario	52 (48.6%)
	Alberta	10 (9.3%)
	British Columbia	16 (14.9%)
	Newfoundland	1 (0.9%)
	International:	26 (24.2%)
	Australia	11 (10.3%)
	Kenya	2 (1.8%)
	New Zealand	2 (1.8%)
	United Kingdon	2 (1.8%)
	United States	9 (8.4%)
	Undisclosed	2 (1.8%)
Occupation, No. (%) (n = 107)	Nurse	49 (45.8%)
	Psychologist/graduate level psychology student	22 (20.5%)
	Radiation therapist	8 (7.5%)
	Social worker	10 (9.3%)
	Scientist/researcher	4 (3.8%)
	Other	9 (8.4%)
	Undisclosed	5 (4.7%)
Community setting, No. (%) (n = 89)	Urban	81 (91.0%)
	Rural	6 (6.7%)
	Undisclosed	2 (2.2%)
Years of prostate cancer experience, No. (%) (n = 89)	0-5	60 (67.4%)
	6-10	17 (19.1%)
	11+	10 (11.2%)
	Undisclosed	1 (1.1%)
Years of sexual health experience, No. (%) (n = 89)	0-5	68 (76.4%)
	6-10	13 (14.6%)
	11+	5 (5.6%)
	Undisclosed	3 (3.4%)
Motivation for course, No. (%) (n = 89)	Fill gap in clinical care	21 (23.6%)
	Want formal training	27 (30.3%)
	An area they would like to explore	10 (11.2%)
	Employer suggested/required it	21 (23.6%)
	General interest	9 (10.1%)
	Undisclosed	2 (2.2%)
Interest in course, No. (%) (n = 89)	Content	54 (60.7%)
	Delivery	5 (5.6%)
	Both	17 (19.1%)
	Not specified	13 (14.6%)
Management support, No. (%) (n = 89)	Yes	80 (89.9%)
	Neither supportive nor unsupportive	6 (6.7%)
	No	2 (2.2%)
	Not specified	1 (1.1%)
3**-**mo follow-up occupation, No. (%) (n = 18)	Nurse	9 (50.0%)
	Psychologist/graduate level psychology student	5 (27.8%)
	Radiation Therapist	1 (5.5%)
	Social Worker	2 (11.1%)
	Other	1 (5.5%)

### Knowledge

**Figure 1:**
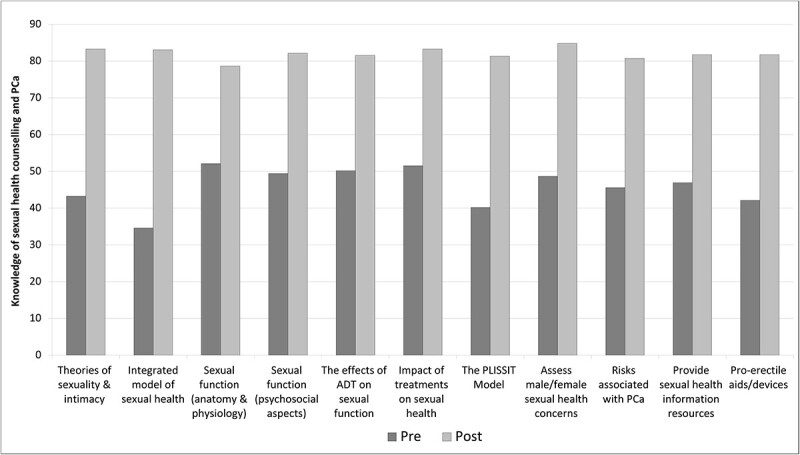
Mean scores on individual items assessing knowledge of sexual health counseling and prostate cancer (PCa).

**Figure 2:**
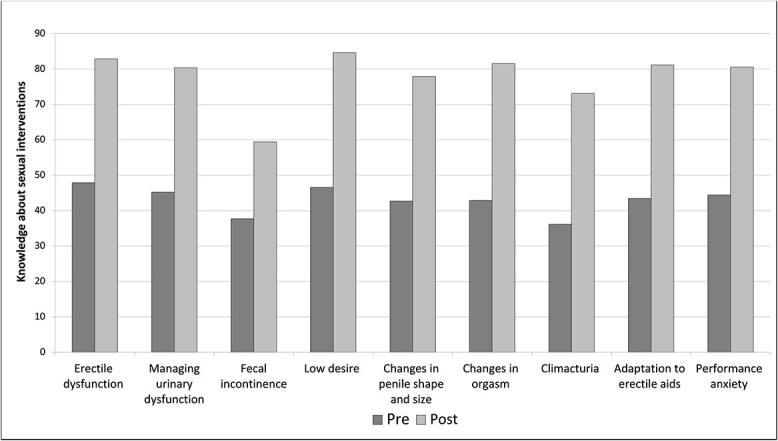
Mean score of individual items assessing knowledge about sexual interventions.

Based on the KSE survey results, the course improved HCP perceived knowledge in all domains. Paired-sample *t*-test analyses demonstrated significantly improved scores in all knowledge domains. Mean (SD) values, *t*-scores, and *P* values are presented in Table 3. Figure 1 shows mean scores on individual items assessing knowledge of sexual health counseling and PCa (see Figure 1). Figure 2 illustrates the mean scores of individual items assessing knowledge about sexual interventions (see Figure 2).

**Table 3:** Mean (SD) scores, *t*-scores, and *P* values of participant KSE survey.

**Domain**	**Measure**	**Precourse, mean (SD)**	**Postcourse, mean (SD)**	**Pre-post t(57), mean**	**Pre-post *P*-value, SD**
Knowledge (n = 58)	Sexual health counseling and PCa	452.41 (239.01)	808.97 (144.76)	10.33	.00
	Intervening with sexual issues	346.90 (219.72)	628.97 (124.23)	9.57	.00
	Intervening with sexual issues for female/male partners	553.45 (316.41)	971.03 (152.14)	10.57	.00
	Collaborating and adjusting counseling	347.93 (163.44)	566.55 (94.99)	11.73	.00
Self-efficacy (n = 58)	Initiating sexual health conversation and providing information resources	178.45 (119.84)	307.93 (97.45)	10.05	.00
	Intervening with sexual issues	351.21 (247.25)	637.24 (213.73)	10.42	.00
	Intervening with sexual issues for female/male partners	515.34 (353.02)	868.10 (290.10)	9.66	.00
	Collaborating and adjusting counseling	230.34 (140.61)	379.83 (125.00)	9.86	.00

Abbreviation: KSE, Knowledge and Self-Efficacy Survey.

### Self-efficacy

**Figure 3:**
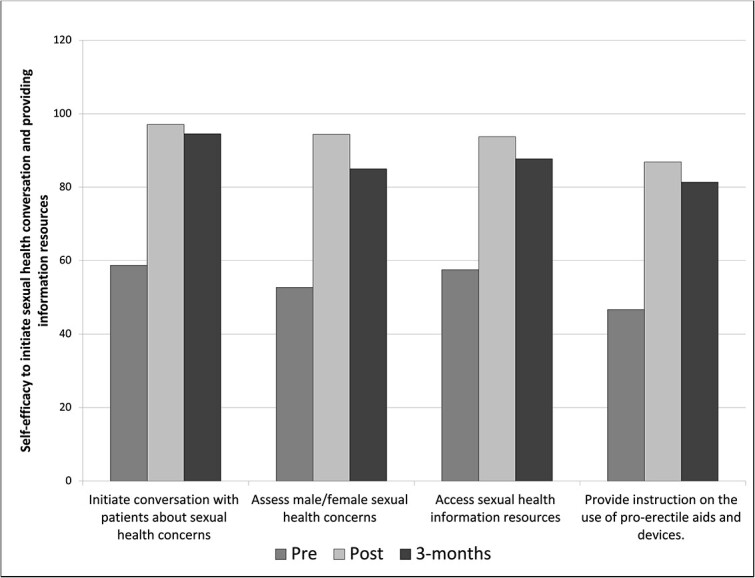
Mean score of individual items on self-efficacy to initiate sexual health conversation and providing information resources.

**Figure 4:**
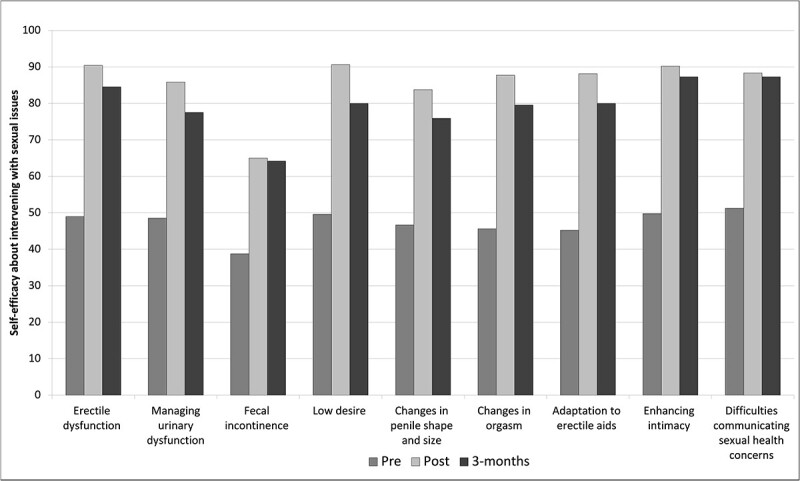
Mean score of individual items on self-efficacy about intervening with sexual issues.

Self-efficacy levels improved from the KSE survey in all domains. Paired-sample *t*-tests demonstrated significant improvements in all self-efficacy domains. The mean scores, standard deviations, t-scores, and p-values are presented in Table 3, as mention in the previous paragraph. As shown in Figure 3, the mean score of individual items on self-efficacy to initiate sexual health conversations and provide information resources increased significantly. Similarly, Figure 4 illustrates the mean score of individual items on self-efficacy regarding interventions with sexual issues.

### Satisfaction

Almost all participants who finished the KSE survey (n = 57) also participated in the satisfaction survey, and 98.2% indicated that they were very satisfied with the SHAReTraining program. Additionally, most respondents (94.7%) agreed that they would recommend the course to colleagues. The SHAReTraining facilitators received praise as well; with most (96.4%) agreeing that the instructors were effective group facilitators.

## Discussion

The SHAReTraining program was developed to increase the knowledge and expertise of Canadian HCPs in addressing sexual dysfunction in patients undergoing PCa treatment. In this study we aimed to evaluate the effectiveness of the SHAReTraining program for enhanceing the perceived knowledge and self-efficacy of HCPs in providing sexual health counseling to PCa patients and their partners. While not a traditional research study, this investigation indicates that SHAReTraining fills a critical gap by enhancing HCP capabilities to manage sexual dysfunction among PCa patients.

Results from the KSE survey revealed that participants entered the course with moderate levels of knowledge and self-efficacy on the topic of sexual dysfunction in PCa patients. However, after completing the 12-week course curriculum, the perceived knowledge and self-efficacy of the participants increased significantly, demonstrating the value of SHAReTraining. Furthermore, findings from the 3-month follow-up survey showed that participant self-efficacy levels remained high over time.

The results from this study are in line with the evaluation by Sung and Lins (2013) of the effectiveness of a 12-week in-person sexual healthcare education program for nursing students.^25^ These authors reported that their program improved knowledge and self-efficacy. Furthermore, research has shown that regardless of course load or modality, sexual education programs for HCP enhance their ability to address patients’ sexual issues.^26^^,^^27^ Overall, sexual health educational initiatives improve the ability, comfort level, and frequency with which HCPs communicate about sexual health with patients.^27^

Despite the potential benefits for both HCP and patients, there has been limited research and development in this underserved area.^26^^,^^27^ Existing sexual health programs typically provide only general insights into sexual health and lack in-depth insights on specific health conditions affecting sexual health. Programs like SHAReTraining contribute to the field by offering a comprehensive and specifically tailored courses focused on PCa-related sexual health issues. SHAReTraining may be more appealing to HCPs who seek to provide comprehensive care to their patients but are not interested in general sexual health training. The dearth of research on sexual health educational interventions for HCPs highlights the potential for programs like SHAReTraining to improve the quality of care and support for PCa patients and their partners.

The SHAReTraining program was designed to provide learning materials and outcomes in a productive and efficient online format to accommodate the busy lives of HCPs. Both the online delivery and the compacted learning time (1.5 hours per week) aligned with HCP preference for internet-based learning and accommodated busy work schedules.

Insufficient knowledge and self-efficacy in HCPs supporting patients dealing with sexual dysfunction following PCa treatment is a global issue. For instance, Dyer et al. (2019) noted that HCPs in the United Kingdom who cared for PCa patients could benefit from opportunities for improved education and clear guidelines regarding erectile dysfunction guidelines.^28^ Wang et al. (2022) also noted that in China, sexual health was underaddressed and suggested that Chinese HCPs could benefit from systematic training on sexual issues.^29^ The online format of SHAReTraining can potentially address these issues by reaching HCPs on a larger, international scale. Despite international time differences relating to the facilitation of the SHAReTraining program in Canada, as well as the delivery of the program in English only, the program was still attractive to international participants (n = 26, 24.2%). This positive result demonstrates the capability of SHAReTraining to serve HCPs from varying geographical backgrounds.

SHAReTraining focuses on helping participants develop the knowledge and skills required to conduct assessments and deliver interventions for common sexual health issues that arise in the care of patients with PCa and their partners. The course utilizes a biopsychosocial approach developed by a multidisciplinary team, making it relevant for all HCPs. As a result, SHAReTraining is a suitable training program for HCPs from diverse backgrounds that effectively equips HCPs with the skills and knowledge needed to deliver sexual health support to patients and their partners coping with PCa treatment.

### Limitations

There are several limitations to this study that should be considered. The single-arm design of this evaluation means that there was no control group available for comparison. The absence of a control group prevents the researchers from attributing the score improvements solely to the SHAReTraining course. Moreover, the study design makes it susceptible to the placebo effect, as participants were self-selected and might have had expectations that the course would enhance their knowledge.

Additionally, it is important to note that the study surveys did not assess the actual knowledge of the participants; rather, the HCPs relied on self-reports to determine perceived knowledge levels. This approach has inherent limitations as retrospective knowledge recall can be less accurate and responses may have been influenced by the postcourse knowledge of the participants. Furthermore, improvements in participant self-efficacy may have led them to overreport their knowledge levels. Future research can address this issue by incorporating a knowledge-based assessment that examines participant knowledge levels before and after the course. Such assessments would provide an objective measure of knowledge acquisition from the course, as opposed to the current method that relies on the self-reported perceived knowledge of the participants.

Furthermore, the rigor of this study could be enhanced by conducting a randomized control trial with purposeful sampling and a waitlist control. This design would add further confidence to the results presented in this article, allowing for a more robust evaluation of the program’s effectiveness.

Furthermore, the 3-month follow-up survey was only administered to the first 3 cohorts, with just 18 participants completing it. This relatively small sample size and short follow-up duration limits our ability to truly assess long-term self-efficacy levels. To address this in future studies, extended follow-up periods should be implemented, with 12 months being the recommended duration, and the follow-up survey should be extended to all course participants. This approach would provide a more comprehensive understanding of the prolonged impacts of virtual training programs like SHAReTraining on the HCP participants’ self-efficacy.

Last, although participants reported increased perceived knowledge and self-efficacy, this study did not assess the impact of SHAReTraining on HCP practices with their patients and partners. However, many SHAReTraining participants went on to become sexual health coaches in the corresponding intervention, the Sexual Health and Rehabilitation eClinic (SHAReClinic),^30^ which provides the research team with an opportunity to examine the impact of the course on HCP competence in serving patients with sexual dysfunction.

## Conclusion

The SHAReTraining program stands as an innovative program that enables HCPs to undergo specialized education in sexual health to serve PCa patients and their partners dealing with sexual dysfunction. The course addresses HCPs’ lack of knowledge while also improving their self-efficacy for initiating and navigating conversations with patients and their partners. The high satisfaction ratings from participants and support from employers for their employees to attend the training underscore the value of the program. The online nature of the course means it is widely accessible to HCPs across Canada, with the potential to expand internationally. It is essential to recognize that SHAReTraining operates within real-world conditions, which contributes to its authenticity and relevance. As such, SHAReTraining is a valuable resource for HCPs seeking specialized training in sexual healthcare for PCa patients and their partners.

## Data Availability

The data presented in this study are available on request from the corresponding author.
